# Determinants of intensive insulin therapeutic regimens in patients with type 1 diabetes: data from a nationwide multicenter survey in Brazil

**DOI:** 10.1186/1758-5996-6-67

**Published:** 2014-05-31

**Authors:** Marilia Brito Gomes, Carlos Antonio Negrato, Roberta Cobas, Lucianne Righeti Monteiro Tannus, Paolla Ribeiro Gonçalves, Pedro Carlos Barreto da Silva, João Regis Ivar Carneiro, Alessandra Saldanha Mattos Matheus, Sergio Atala Dib, Mirela Jobim Azevedo, Márcia Nery, Melanie Rodacki, Lenita Zajdenverg, Renan Magalhães Montenegro Junior, Janice Sepulveda, Luis Eduardo Calliari, Deborah Jezini, Neuza Braga, Jorge L Luescher, Renata S Berardo, Maria Carmo Arruda-Marques, Renata M Noronha, Thais D Manna, Roberta Salvodelli, Fernanda G Penha, Milton C Foss, Maria C Foss-Freitas, Antonio C Pires, Fernando C Robles, Maria de Fátima S Guedes, Patricia Dualib, Saulo C Silva, Emerson Sampaio, Rosangela Rea, Ana Cristina R Faria, Balduino Tschiedel, Suzana Lavigne, Luis Henrique Canani, Alessandra T Zucatti, Marisa Helena C Coral, Daniela Aline Pereira, Luiz Antonio Araujo, Monica Tolentino, Hermelinda C Pedrosa, Flaviane A Prado, Nelson Rassi, Leticia B Araujo, Reine Marie C Fonseca, Alexis D Guedes, Odelissa S Matos, Catia C Palma, Rossana Azulay, Adriana C Forti, Cristina Façanha, Ana Paula Montenegro, Naira H Melo, Karla F Rezende, Alberto Ramos, João Soares Felicio, Flavia M Santos

**Affiliations:** 1 Unit of Diabetes, Universidade Estadual do Rio de Janeiro, Avenida 28 de Setembro, 77, 3 andar CEP 20.551-030 Rio de Janeiro, Brazil; 2 Bauru’s Diabetics Association, Bauru, São Paulo, Brazil; 3 Diabetes Unit, Federal University of São Paulo State, São Paulo, Brazil; 4 Federal University Hospital of Porto Alegre, Rio Grande do Sul, Brazil; 5 Diabetes Unit, University Hospital of São Paulo, São Paulo, Brazil; 6 Federal University of Rio de Janeiro, Rio de Janeiro, Brazil; 7 Federal University of Ceará, Ceará, Brazil; 8 Santa Casa Misericórdia, Belo Horizonte, Minas Gerais, Brazil; 9 Santa Casa Misericórdia São Paulo, São Paulo, Brazil; 10 Universidade Federal Amazonas, Manaus, Brazil; 11 Hospital Geral de Bonsucesso, Rio de Janeiro, Brazil; 12 Hospital Universitário Clementino Fraga Filho – IPPMG, Rio de Janeiro, Brazil; 13 University Hospital of São Paulo, São Paulo, Brazil; 14 Faculdade de Ciências Médicas da Santa Casa de São Paulo, São Paulo, Brazil; 15 Instituto da Criança do Hospital das Clínicas da Universidade de São Paulo, São Paulo, Brazil; 16 Hospital das Clínicas da Faculdade de Medicina de Ribeirão Preto – USP, Ribeirão Preto, Brazil; 17 Ambulatório da Faculdade Estadual de Medicina de São José do Rio Preto, Ribeirão Preto, Brazil; 18 Centro de Diabetes da Escola Paulista de Medicina, Ribeirão Preto, Brazil; 19 Clínica de Endocrinologia da Santa Casa de Belo Horizonte, Belo Horizonte, Brazil; 20 Universidade Estadual de Londrina, Londrina, Brazil; 21 Hospital de Clínicas da Universidade Federal do Paraná, Porto Alegre, Brazil; 22 Instituto da Criança com Diabete Rio Grande Sul, Rio Grande do Sul, Brazil; 23 Instituto da Criança com Diabetes, Grupo Hospitalar Conceição, Porto Alegre, RS, Brazil; 24 Hospital Universitário de Santa Catarina, Florianópolis, Brazil; 25 Instituto de Diabetes-Endocrinologia de Joinville, Joinville, Brazil; 26 Hospital Regional de Taguatinga, Brasília, Brasilia, Brazil; 27 Hospital Geral de Goiânia, Goiânia, Brazil; 28 Centro de Diabetes e Endocrinologia do Estado da Bahia, Goiânia, Brazil; 29 Universidade Federal do Maranhão, São Luís, Brazil; 30 Centro Integrado de Diabetes e Hipertensão do Ceará, Fortaleza, Brazil; 31 Universidade Federal de Sergipe, Aracaju, Brazil; 32 Hospital Universitário Alcides Carneiro, Campina Grande, Brazil; 33 Hospital Universitário João de Barros Barreto, Pará, Belém, Brazil

**Keywords:** Type 1 diabetes, Chronic complications, Insulin regimens, Brazil

## Abstract

**Background:**

To evaluate the determinants of intensive insulin regimens (ITs) in patients with type 1 diabetes (T1D).

**Methods:**

This multicenter study was conducted between December 2008 and December 2010 in 28 public clinics in 20 Brazilian cities. Data were obtained from 3,591 patients (56.0% female, 57.1% Caucasian). Insulin regimens were classified as follows: group 1, conventional therapy (CT) (intermediate human insulin, one to two injections daily); group 2 (three or more insulin injections of intermediate plus regular human insulin); group 3 (three or more insulin injections of intermediate human insulin plus short-acting insulin analogues); group 4, basal-bolus (one or two insulin injections of long-acting plus short-acting insulin analogues or regular insulin); and group 5, basal-bolus with continuous subcutaneous insulin infusion (CSII). Groups 2 to 5 were considered IT groups.

**Results:**

We obtained complete data from 2,961 patients. Combined intermediate plus regular human insulin was the most used therapeutic regimen. CSII was used by 37 (1.2%) patients and IT by 2,669 (90.2%) patients. More patients on IT performed self-monitoring of blood glucose and were treated at the tertiary care level compared to CT patients (p < 0.001). The majority of patients from all groups had HbA1c levels above the target. Overweight or obesity was not associated with insulin regimen. Logistic regression analysis showed that economic status, age, ethnicity, and level of care were associated with IT (p < 0.001).

**Conclusions:**

Given the prevalence of intensive treatment for T1D in Brazil, more effective therapeutic strategies are needed for long term-health benefits.

## Introduction

The incidence of T1D has increased in recent decades in developed and developing countries, such as Brazil [1,2]. T1D negatively impacts quality and duration of life, due to morbidity and mortality from chronic complications which increase the costs of medical care [3-5]. A steady decline in mortality rates due to end-stage renal disease and a steady increase in mortality from cardiovascular disease (CVD) have been observed in recent decades [6,7].

The Diabetes Control and Complications Trial (DCCT) and its long-term follow-up study, the Epidemiology of Diabetes Interventions and Complications (EDIC) Trial, suggested that the mechanisms that underlie the accelerated atherosclerosis observed in this population are associated with poor glycemic control [8-10]. However, other factors related to CVD may contribute to atherosclerosis in patients with T1D, such as oxidative stress [11], markers of insulin resistance [12], markers of low-grade inflammation [13], dyslipidemia [14], hypertension [14,15], and obesity [15]. These findings support the importance of optimizing glycemic control and CV risk factors to reduce the risk of diabetes-related complications. However, the few observational studies that have been conducted with these patients have demonstrated the difficulty of achieving glycemic control and CV risk factor targets despite treatment with multiple daily insulin injections or continuous subcutaneous insulin infusion (CSII) using a pump [9,15,16].

The SEARCH for Diabetes in Youth Study demonstrated poor glycemic control, (HbA1c >9.5%), in 17.0% of these patients and elevated blood pressure in 5.9% [17,18] emphasizing the difficulty of treating these patients in daily clinical practice.

The Brazilian Type 1 diabetes Study Group (BrazDiab1SG) conducted a survey that analyzed the demographic, clinical, and economic data of patients with T1D who received medical care at public clinics in Brazil. The absence of Brazilian national data on the determinants of clinical intensive insulin therapeutic regimen (IT) used by patients with T1D led the BrazDiab1SG to conduct this study to provide current and reliable data on this topic.

## Research design and methods

### Overview of the Brazilian type 1 diabetes study group

BrazDiab1SG, a retrospective, cross-sectional, multicenter observational study, was conducted from December 2008 to 2010 in 28 public secondary and tertiary care clinics located in 20 cities in four Brazilian geographic regions (north/northeast, mid-west, southeast, and south). The data collection methods have been described previously [19-21]. The inclusion criteria consisted of patients with a diagnosis of T1D by a physician. All of the patients received health care and had all the expenses with the treatment covered by the National Brazilian Health Care System and all of the eligible participating centers had to have a diabetes clinic with at least one endocrinologist and had to provide data from a minimum of 50 consecutive outpatients who regularly attended the clinic. A total of 3,591 patients who met these criteria were included in the study. All patients were diagnosed between 1960 and 2010. Patients were excluded if they did not fulfill all of the inclusion criteria.

Each center’s local ethics committee approved the study (Appendix 1). Written informed consent was obtained from all of the patients or their parents.

### Data collection

We collected demographic and economic status data in an interview (by a questionnaire) during a clinical visit. The following variables were also recorded: current age; age at diagnosis; duration of diabetes; blood pressure; insulin therapeutic regimen; frequency of daily self-monitoring of blood glucose (SMBG); comorbidities; self-reported frequency of severe hypoglycemia; hospitalization due to diabetic ketoacidosis or hyperglycemia and smoking status (current smoking was defined as smoking more than one cigarette per day at the time of the interview). Patients younger than 13 years were considered children, patients between 13 and 19 years old were classified as adolescents, and patients older than 19 were considered adults.

The levels of HbA1c, fasting plasma glucose (FPG), total cholesterol, LDL cholesterol, HDL cholesterol, and triglycerides measured during the last clinical visit were obtained from the patients’ medical records. Within one year of the study assessment, patients with a diabetes duration greater than or equal to five years from diagnosis were screened for the following chronic diabetes-related complications: retinopathy (classified as absent, nonproliferative, or proliferative; by fundoscopy); clinical nephropathy (according to American Diabetes Association (ADA) recommendations [22]); macrovascular diseases (classified as clinical coronary artery disease, stroke, and peripheral vascular disease); and foot pathologies.

The following ADA goals for adequate metabolic and clinical control [22] were adopted by the BrazDiab1SG. Good glycemic control was defined as HbA1c <58 mmol/mol (7.5%) for patients with T1D between 13 and 19 years old; <64 mmol/mol (8%) for patients between 6 and 12 years old; between 58 mmol/mol (7.5%) and 69 mmol/mol (8.5%) for patients <6 years old; and <53 mmol/mol (7%) for adult patients with T1D [22]. Poor glycemic control was defined as HbA1c higher than 75 mmol/mol (9%).

In adults, overweight was defined as a BMI ≥25 kg/m^2^, and obesity as a BMI ≥30 kg/m^2^[15]. In children and adolescents, overweight was defined as a BMI ≥85th percentile for age and gender, and obesity as a BMI ≥95th percentile for age and gender [23].

HbA1c values obtained during the last clinical visit and the measurement methods were collected from the patients’ medical charts. HbA1c measurements were obtained for 3,097 patients (86.2%) using methods certified by the National Glycohemoglobin Standardization Program (NGSP). Of the total population, 1,652 patients (53.2%) were evaluated using high-performance liquid chromatography, whereas 1,445 patients (46.7%) were evaluated using turbidimetry. The HbA1c measurements using methods not certified by the NGSP, missing data, and HbA1c measurements obtained more than one year before the study assessment were excluded from the glycemic control analyses (n = 494; 13.8%). FPG, triglycerides, HDL cholesterol, and total cholesterol were measured using enzymatic techniques . LDL cholesterol level was calculated using Friedewald’s equation [24].

### Insulin therapeutic regimens

Insulin therapeutic regimens were performed according to the center where patients were treated and to the guidelines from Brazilian Diabetes Society (BDS) [25] classified into the following five groups: group 1, conventional therapy (CT) (intermediate human insulin, one to two injections daily); groups 2 to 4, patients using three or more insulin injections daily as follows: group 2, one or two injections of intermediate human insulin plus regular human insulin; group 3, one or two insulin injections of intermediate human insulin plus short-acting insulin analogues; group 4, basal-bolus (one or two insulin injections of long-acting insulin analogues plus short-acting insulin analogues or long-acting insulin-analogues plus regular insulin); and group 5, basal-bolus with CSII. Groups 2 to 5 were considered IT groups.

### Sample calculation and economic status

The study sample represented the distribution of T1D cases across four geographic regions in Brazil. This distribution was estimated using the overall population distribution reported in the 2000 Brazilian Institute of Geography and Statistics Population Census (IBGE) [26]. These data were combined with the national estimates of diabetes prevalence, which were derived from a 1988 survey, to determine the minimum number of patients to be studied in each region [27]. Patient economic status was defined according to the Brazilian Economic Classification Criteria [28]. This classification considers educational level, which is categorized as illiterate/incomplete primary education, complete primary education/incomplete secondary education, complete secondary education/incomplete high school, complete high school/some college, or complete college education. The following economic status classifications were considered for this analysis: high, middle, low, and very low [28].

### Statistical analysis

The data are presented as mean (±SD) values for continuous variables and as counts (relative frequencies) for discrete variables. When multiple comparisons were used, we used the Bonferroni correction. A multivariate logistic regression model was performed with IT (yes for groups 2 to 5 and no for CT) as the dependent (outcome) variable and demographic data, such as gender, age (stratified as children, adolescents, and adults), economic status, self-reported ethnicity (Caucasian or non-Caucasian), and duration of diabetes, as independent (exposure) variables. All of the analyses were performed using the Statistical Package for the Social Sciences (SPSS version 17.0, SPSS, Inc., Chicago, IL, USA). Odds ratios (ORs) with 95% confidence intervals (CIs) were calculated when indicated. A two-sided p < 0.05 was considered statistically significant.

## Results

### Overview of participant demographics, socioeconomic status, and level of care

Data were obtained from 2,961 patients [excluded: n = 630 (17.6%): 494 because of missing HbA1c data and 136 because of missing data about insulin therapeutic regimens]. The median follow-up time of the 28 centers was 4.2 years (<1 to 49). The majority of the patients attended tertiary care centers. We observed that more patients from groups 3 and 4 had visited the clinic in the year before the beginning of the study. The economic status of 1,977 (69.0%) patients was either very low or low. The demographic data of the studied population are shown in Table 1.

**Table 1:** Demographic data of the study population

**Variable**	**Value**
Age, y	19 (1-66)
Gender, F (%)	2,010 (56.0)
Age at diagnosis, y	10.0 (<1 to 44)
Age at diagnosis, y (%)	
0-4.9	667 (18.5)
5-9.9	961 (26.8)
10-14.9	941 (26.2)
≥15	1,022 (28.5)
Ethnicity, n (%)	
Caucasian	2,049 (57.1)
Non-Caucasian	1,542 (42.9)
Economic status*	
High	245 (7.1)
Medium	771 (22.4)
Low	1,163 (33.9)
Very low	1,255 (36.6)
Duration of diabetes, y	7.0 (<1 to 50)
Geographic region (%)	
Southeast	1,424 (39.7)
North/northeast	1,113 (31)
South	820 (22.8)
Mid-west	234 (6.5)
Level of care, n (%)	
Secondary	995 (27.7)
Tertiary	2,596 (72.3)

y, year; F, female; data are presented as n (%) or median (minimum/maximum).

*Data available for 3,434 patients; **data available for 3,553 patients.

### Overview of diabetes treatment modality, glycemic control, and coexistence of acute and chronic diabetes-related complications

Combined intermediate plus regular human insulin was the most frequently used insulin therapeutic regimen. IT (groups 2 to 5) was being administered to 2,669 patients (90.2%). More patients who received IT attended tertiary care centers compared to patients who received CT (p < 0.001).

Overall, the patients from group 1 were older, had been diagnosed when they were older, were more frequently non-Caucasians, and more frequently had a low or very low socioeconomic status compared to the patients from each individual group who received IT.

Three or more daily injections of regular human insulin or short-acting insulin analogues were used by 1,766 (57.1%) patients, and three or more SMBG daily tests were reported by 2,041 (65.9%) patients. A higher daily number of SMBG tests was observed in patients who received IT (groups 2 to 5) than in the patients who received CT (group 1) (p < 0.001).

The lowest total daily insulin dose/kilogram was noted in groups 1 and 5 compared to the other groups (p < 0.001). Considering the total insulin dose the percentage of doses (bolus) of short-acting in relation to long-acting insulin analogues for patients from group 4 was greater than the percentage of doses ( bolus ) of regular or short-acting insulin analogues in relation to intermediate insulin in groups 2 and 3, respectively (p < 0.001).

Group 5 [8.3 ± 1.6% (67.7 ± 17.6 mmol/mol)] had lower HbA1c compared to patients from group 1 [9.2 ± 2.5% (77.1.0 ± 27.4 mmol/mol)], group 2 [9.5 ± 2.4% (81.0 ± 26.9 mmol/mol)], and group 3 [9.2 ± 2.3% (77.0 ± 25.3 mmol/mol)] (p < 0.01 for each). No difference in HbA1c was noted between groups 4 and 5 or between groups 1 and 2. A higher proportion of patients from groups 1 and 4 compared to patients from group 2 reached the HbA1c target (p < 0.005). A higher proportion of patients from group 2 had poor glycemic control compared to patients from the other groups (p < 0.001).

The use of an insulin regimen was related with the frequency of severe hypoglycemia and the frequency of hospitalization due to hyperglycemia or ketoacidosis (ANOVA; p < 0.001), but no association reached statistical significance after correction for multiple comparisons. The frequency of any microvascular complication was lower in group 3 compared to the other groups (p < 0.006). Patients from group 5 used more antihypertensive drugs than the other groups (p < 0.05). No difference was found regarding the use of antidyslipidemic drugs among all the groups. All these data are shown in Table 2.

**Table 2:** Clinical and laboratory data according to insulin regimen

	**Intermediate human insulin**			**Long acting plus short acting or regular**	*CS II	**P value**
	**Monotherapy**	**Plus regular**	**Plus short acting**			
	**Group 1**	**Group 2**	**Group 3**	**Group 4**	**Group 5**	
Demographic and economic status data						
n	249	1475	627	573	37	
Gender F (%)	135 (54.4)	828 (56.2)	371 (59.2)	532 (57.9)	25 (67.6)	0.4
Level of care (tertiary)	138 (55.2)	1020 (69.2)	570 (90.9)	444 (77.5)	30 (81.1)	< 0.001
Clinical visits in the previous year	3.8 ± 1.6	4.1 ± 1.6	4.5 ± 1.4	4.2 ± 1.5	4.5 ± 1.6	<0.001
Age, y	26.5 ± 14.5	21.2 ± 10.9	19.3 ± 11.5	21.7 ± 12.4	22.7 ± 9.9	0.001
Duration of DM ,y	10.6 ± 9.8	9.3 ± 7.5	9.6 ± 8.2	10.8 ± 8.5	12.3 ± 7.0	0.001
Age at diagnosis, y	15.8 ± 9.6	11.7 ± 7.7	9.6 ± 7.5	10.9 ± 7.7	10.4 ± 7.3	< 0.001
Ethinicity, y(%)**						< 0.001
Caucasian	113 (45)	754 (51.1)	448 (71.5)	414 (72.3)	29 (78.4)	
Non caucasian	136 (54.4)	720 (48.9)	179 (28.5)	159 (27.7)	8 (21.6)	
Economic status (%)**						
High	13 (5.5)	66 (4.5)	42 (7.0)	85 (15.5)	5 (14.3)	
Medium	30 (12.8)	265 (18.3)	192 (32.2)	169 (30.8)	19 (54.3)	
Low	68 (28.9)	515 (35.6)	214 (35.9)	180 (32.8)	11(31.1)	
Very low	125 (52.8)	601 (41.6)	148 (24.8)	115 (20.9)	0	
Glycemic control and insulin dose						
A1c (%)	9.2 ± 2.5	9.5 ± 2.4	9.2 ± 2.3	8.8 ± 2.0	8.3 ± 1.6	<0.001
A1c (mmol/mol)	77.1 ± 27.4	81.0 ± 26.9	77.0 ± 25.3	72.8 ± 22.4	67.7 ± 17.6	<0.001
A1c (good) n(%)	58 (23.4)	219 (14.9)	118 (18.8)	123(21.5)	9(24.3)	<0.001
A1c (poor) n (%)	114 (46.0)	795 (54)	281 (44.8)	209 (36.5)	9 (24.3)	<0.001
SMBG/daily	2.5 ± 1.7	2.9 ± 1.6	3.7 ± 1.4	4.0 ± 1.5	5.2 ± 1.6	<0.001
Insulin dose(U/Kg/day)	0.6 ± 0.3	0.9 ± 0.4	0.9 ± 0.3	0.8 ± 0.3	0.7 ± 0.3	0.001
Bolus (%)	_	21.8 ± 11.6	24.5 ± 12.7	33.2 ± 13.9	_	<0.001
Cardiovascular risk factors						
sBP (mmHg)	113.8 ± 18.8	110.6 ± 17.3	110.4 ± 15.9	110.9 ± 16.9	116.0 ± 15.3	0.2
dBP (mmHg)	73.5 ± 11.9	71.0 ± 11.5	69.8 ± 10.9	71.2 ± 11.4	72.4 ± 9.1	0.09
Cholesterol (mg/dl)	171.0 ± 43.9	172.7 ± 43.5	165.2 ± 33.9	167.1 ± 37.8	176.8 ± 53.6	< 0.001
Triglycerides (mg/dl)	102.7 ± 90.4	99.1 ± 79.8	78.6 ± 44.5	84.1 ± 55.6	81.2 ± 58.7	< 0.001
HDL cholesterol (mg/dl)	49.8 ± 12.1	51.7 ± 4.5	54.6 ± 13.9	55.6 ± 15.8	63.9 ± 25.3	< 0.001
Non HDL cholesterol (mg/dl)	121.7 ± 43.1	121.0 ± 41.6	110.5 ± 31.5	112.3 ± 34.1	112.7 ± 40.4	< 0.001
LDL-Cholesterol	102.1 ± 35.7	102.3 ± 34.0	95.3 ± 28.0	95.9 ± 30.1	96.6 ± 32.6	< 0.001
BMI (kg/m2)	21.8 ± 4.9	22.0 ± 4.3	21.5 ± 4.1	21.4 ± 3.8	22.6 ± 3.6	0.02
Overweight or obese n(%)	71(28.5)	481 (32.8)	220 (35.1)	164(28.7)	13 (35.1)	0.2
Acute and chronic complications						
Severe hypoglycemic, yes (%)	15 (13.3)	157 (18.3)	61 (13.4)	81 (19.4)	3 (8.8)	<0.02
Hospitalizations****,yes (%)	41 (16.5)	226 (15.3)	65 (10.4)	66 (11.5)	2 (5.4)	0.004
Microvascular complications, yes (%)	55 (33.7)	259 (26.0)	100 (22.6)	138 (33.5)	14 (43.8)	<0.001

The data are presented as counts (percentage), means ± SD or medians (minimum/maximum).

*CSII continuous insulin infusion; * *African-Brazilians, Mulattos, Asians, Native Indians; ***Data were available for 3,434 patients.

****Hospitalization considered for diabetic ketoacidosis or hyperglycemia.

Using multivariate analysis, the adjusted ORs for IT use showed that the most important variables were level of care, age, economic status, and ethnicity. The adjusted model is shown in Table 3.

**Table 3:** Final adjusted logistic regression model for intensive insulin therapeutic regimen use and demographic and economic status of the study population

	**n**	**OR (95% CI)**	**Adjusted p-value**
			
Level of care		-	
Secondary	759	0.39 (0.53-0.71)	<0.001
Tertiary	2,202	-	*Reference*
Economic status*			
Very low	989	-	*Reference*
High	211	1.52 (2.82-5.20)	0.001
Medium	675	2.19 (3.37-5.19)	<0.001
Low	988	1.43 (1.98-2.75)	<0.001
Age	1,488	-	*Reference*
Adults			
Adolescents	790	2.53 (3.79-5.68)	<0.001
Children	683	1.74 (2.54-3.71)	<0.001
Ethnicity	1,758	1.12 (1.49-1.98)	<0.001
Caucasian			
Non-Caucasian†	1,203		*Reference*

*Data available for 2,863 patients; †African-Brazilians, Mulattos, Asians, Native Indians.

### Overview of frequency of cardiovascular risk factors

Overweight or obesity was observed in 978 patients (31.6%), and weight status was not statistically related with the type of insulin regimen. No difference was observed in systolic blood pressure between the insulin therapeutic regimen groups. The patients from group 1 presented higher diastolic blood pressure (73.5 ± 11.9 mmHg), higher triglycerides (102.7 ± 90.4 mg/dl), lower HDL cholesterol (49.8 ± 12.1 mg/dl), higher non-HDL cholesterol (121.7 ± 43.1 mg/dl), and higher LDL cholesterol (111.1 ± 35.7 mg/dl) than the patients from the other groups (p < 0.001). Group 2 presented higher total cholesterol than the other groups (172.7 ± 43.5) (p < 0.001). These data are shown in Table 2.

## Discussion

The evaluation of IT determinants in Brazilian patients with T1D revealed that approximately 90.0% of the patients with T1D who were treated by an endocrinologist received ITs. The majority of these patients were treated at tertiary care centers (university hospitals) and were using intermediate plus regular human insulin or short-acting insulin analogues with a lower bolus dose of short-acting or regular insulin. Fewer than 20% of the patients used long-acting plus short-acting insulin analogues, and few patients were using CSII. The use of IT was correlated with age, economic status, ethnicity, and level of care. In all of the insulin therapeutic regimen groups, HbA1c was above the target level in more than 70.0% of the patients. Additionally, overweight and obesity, which were found in approximately 31% of the patients, were not directly associated with any insulin therapeutic regimen. Although hospitalization due to diabetic ketoacidosis or hyperglycemia and self-reported severe hypoglycemia were related with the use of insulin therapeutic regimens, no statistically significant difference in those frequencies was observed between regimens. Our results suggest that it is necessary to improve diabetes management and optimize insulin therapeutic regimens in patients with T1D in Brazil to achieve HbA1c levels within the established targets.

The results of DCCT/EDIC have led to proposals for intensive management of T1D to improve glycemic control and avoid or postpone diabetes-related complications [8,9]. However, several observational studies have shown that in daily clinical practice, many challenges, such as social, economic, familial, and psychological factors, are important barriers to intensive management of T1D [17,19-21]. A recent observational study conducted in Germany by Ziegler and colleagues [29] found that an increase in SMBG frequency above five times daily did not result in improved metabolic control, as observed in another study [21]. Ziegler et al. [29] found better glycemic control in patients with T1D who received conventional insulin treatment with three or fewer insulin injections than in patients who received four or more insulin injections per day or CSII.

A similar proportion of our patients who received CT (group 1) achieved the HbA1c targets compared to the patients from groups 4 and 5, although the latter groups had lower mean HbA1c and less patients with poor glycemic control. These findings suggest that an inverse correlation between treatment complexity and glycemic control could exist in several patients, as demonstrated in a recently published meta-analysis [30]. This meta-analysis suggested that adherence is positively correlated with glycemic control in pediatric patients with T1D. The weaker post-DCCT association between adherence and glycemic control suggested that the approach to intensive diabetes management has several shortcomings.

We used the definition of IT that was also used in the SEARCH study, which is the use of at least three insulin injections daily [31]. Although other study have considered an IT to be the use of four or more insulin injections daily or the use of CSII [29], there is no worldwide consensus on this definition. The SEARCH study was a six-center observational study conducted in the USA, in which youths who used CSII exhibited the lowest HbA1c. However, similar to our results, more than 70% of the adolescents, regardless of insulin regimen, had HbA1c that was unacceptably high [32]. That study concluded that there is a need to more fully assess and understand the factors associated with the insulin regimens recommended by providers and the influence of race/ethnicity, education, and socioeconomic status on these treatment recommendations and to develop more effective treatment strategies, particularly for adolescents [32].

In the SEARCH study [32], it was found that CSII was more frequently used by females and patients from high income classes. The small number of patients using CSII in our sample did not allow us to analyze this insulin therapeutic regimen alone, but it is important to emphasize that CSII therapy was the most expensive modality of treatment in our study [3]. However, considering ITs as a whole, our data show similar results concerning economic status, which reflects education level, ethnicity, and age, but not gender. Additionally, in our study, self-reported hypoglycemia, hospitalization, and overweight or obesity were not related to a specific IT, as previously described [32]. Moreover, a trend toward a higher number of SMBG checks performed daily with more intensive insulin therapeutic regimens was also noted, as described in another study [29]. Although an IT is preferred for young patients, the choice of regimen must be individualized based on the youth and the family’s ability to comply with the prescribed plan [29-32]. Considering the cardiovascular risk factors, in general, the CT patients presented the poorest outcomes. Although the patients who received CSII had a higher frequency of microvascular complications and were using more antihypertensive drugs, our cross-sectional study design could not determine whether the prescription of a given IT or antihypertensive drugs was due to an already preexisting diabetes-related complication.

The number of clinical visits was related with the intensity of the insulin therapeutic regimen, which reinforced the idea that regular clinic attendance is an important component of intensive diabetes management [33]. Strategies must be developed to improve accessibility to the clinic and to identify those patients who frequently miss appointments [33].

The strengths of our study include a large sample size that represents the diverse, young Brazilian population with T1D, which includes a wide range of ethnic groups and socioeconomic backgrounds from all geographic regions of the country. The data were collected using a uniform, standardized recruitment protocol in all of the participating centers; therefore, the data represent a large occupational cohort.

Finally, several limitations of our study must be addressed. One limitation was the sample characteristics. All of the patients lived in large cities and were cared for by a specialist in a public health center; thus, patients who relied on primary care facilities and lived in rural areas may not have been considered. However, this group of patients with T1D is the minority of those who receive treatment in Brazil, i.e., fewer than 1%, according to the survey conducted in 1988 [18]. Another limitation was the absence of psychosocial evaluation. Family support and patient self-efficacy have been associated with several positive outcomes, including better glycemic control. Self-reported severe hypoglycemia and the absence of a unique reference laboratory for blood biochemical analysis could be considered other limitations.

## Conclusions

The majority of our patients did not meet the target for HbA1c, independent of insulin therapeutic regimen. Similar number of patients using CT, CSII, and short- and long-acting insulin analogues reached the target HbA1c. High economic status was an important determinant of IT use.

Given the high prevalence of aggressive treatments for T1D currently used in the Brazilian population, more effective therapeutic strategies and diabetes care pathways are needed for long-term health benefits.

## Appendix 1

≠Brazilian Type 1 diabetes Study Group (BrazDiab1SG)

Executive steering committee: Marilia Brito Gomes (chair), and Carlos Antonio Negrato.

**Universidade Estado Rio de Janeiro**: Roberta Cobas*, Alessandra Matheus, Lucianne Tannus; Universidade Federal Rio de Janeiro: Lenita Zajdenverg*, Melanie Rodacki; Hospital Geral de Bonsucesso: Neuza Braga Campos de Araújo*, Hospital Universitário Clementino Fraga Filho – IPPMG: Dr. Jorge Luiz Luescher*; Renata Szundy Berardo; Serviço de Diabetes da Disciplina de Endocrinologia e Metabologia do Hospital das Clínicas da Universidade de São Paulo: Marcia Nery*; Catarina Cani; Maria do Carmo Arruda-Marques; Unidade de Endocrinologia Pediátrica da Santa Casa de Misericórdia de São Paulo: Luiz Eduardo Calliari*, Renata Maria de Noronha; Instituto da Criança do Hospital das Clínicas da Universidade de São Paulo: Thais Della Manna*, Roberta Salvodelli, Fernanda Garcia Penha; Hospital das Clínicas da Faculdade de Medicina de Ribeirão Preto – USP: Milton Cesar Foss*, Maria Cristina Foss-Freitas; Ambulatório da Faculdade Estadual de Medicina de São José do Rio Preto: Antonio Carlos Pires*, Fernando Cesar Robles; Associação de Diabéticos de Bauru: Carlos Antonio Negrato*, Maria de Fatima Guedes; Centro de Diabetes da Escola Paulista de Medicina: Sergio Atala Dib*, Patricia Dualib; Clínica de Endocrinologia da Santa Casa de Belo Horizonte Setor Diabetes Tipo 1: Saulo Cavalcanti da Silva*, Janice Sepulveda; Ambulatório Multiprofissional de Atendimento à Diabetes do Hospital de Clínicas da Universidade Estadual de Londrina: Henriqueta Guidio de Almeida*, Emerson Sampaio; Hospital de Clínicas da Universidade Federal do Paraná: Rosangela Roginski Rea*, Ana Cristina Ravazzani de Almeida Faria; Instituto da Criança com Diabete Rio Grande Sul: Balduino Tschiedel*, Suzana Lavigne, Gustavo Adolfo Cardozo; Hospital de Clínicas de Porto Alegre: Mirela Azevedo*, Luis Henrique Canani, Alessandra Teixeira Zucatti; Hospital Universitário de Santa Catarina: Marisa Helena Cesar Coral*, Daniela Aline Pereira; Instituto de Diabetes-Endocrinologia de Joinville: Luiz Antonio de Araujo*; Hospital Regional de Taguatinga, Brasília: Hermelinda Cordeiro Pedrosa*, Monica Tolentino; Flaviene Alves Prado; Hospital Geral de Goiânia: Dr Alberto Rassi: Nelson Rassi*, Leticia Bretones de Araujo; Centro de Diabetes e Endocrinologia do Estado da Bahia: Reine Marie Chaves Fonseca*; Alexis Dourado Guedes, Odelisa Silva de Mattos; Universidade Federal do Maranhão: Manuel Faria*, Rossana Azulay; Centro Integrado de Diabetes e Hipertensão do Ceará: Adriana Costa e Forti*, Maria Cristina Façanha; Universidade Federal do Ceará: Renan Montenegro Junior*, Ana Paula Montenegro; Universidade Federal de Sergipe: Naira Horta Melo*, Karla Freire Rezende; Hospital Universitário Alcides Carneiro: Alberto Ramos*; Hospital Universitário João de Barros Barreto, Pará: João Soares Felicio*, Flavia Marques Santos; Hospital Universitário Getúlio Vargas, Hospital Adriano Jorge: Deborah Laredo Jezini*.

## Abbreviations

T1D: Type 1 diabetes; CT: Conventional therapy; IT: Intensive insulin regimen; CVD: Cardiovascular disease; DCCT: The diabetes control and complications trial; EDIC: The epidemiology of diabetes interventions and complications trial; CSII: Continuous subcutaneous insulin infusion; HbA1c: Glycated hemoglobin; BrazDiab1SG: Brazilian type 1 diabetes study group; SMBG: Self-monitoring of blood glucose; FBG: Fasting blood glucose; ADA: American diabetes association; BDS: Brazilian diabetes society; NGSP: National glycohemoglobin standardization program; sBP: Systolic blood pressure; dBP: Diastolic blood pressure.

## Competing interests

The authors declare that they have no conflict of interest.

## Authors’ contributions

MBG and CAN contributed equally to analyzing the data and writing the manuscript. ATKS edited the final version. MBG had full access to all study data and was responsible for submitting the manuscript. All authors read and approved the final manuscript.

## References

[B1] The DIAMOND Project Group Incidence and trends of childhood type 1 diabetes worldwide 1990–1999 Diabet Med 2006 23 857 866 16911623 10.1111/j.1464-5491.2006.01925.x

[B2] Negrato CA Dias JP Teixeira MF Dias A Salgado MH Lauris JR Montenegro RM Jr Gomes MB Jovanovic L Temporal trends in incidence of type 1 diabetes between 1986 and 2006 in Brazil J Endocrinol Invest 2010 33 373 377 19620822 10.1007/BF03346606

[B3] Cobas RA Ferraz MB Matheus AS Tannus LR Negrato CA Antonio de Araujo L Dib SA Gomes MB Brazilian Type 1 Diabetes Study Group The cost of type 1 diabetes: a nationwide multicentre study in Brazil Bull World Health Organ 2013 91 434 440 10.2471/BLT.12.110387 24052680 PMC3777141

[B4] Miller RG Secrest AM Sharma RK Songer TJ Orchard TJ Improvements in the life expectancy of type 1 diabetes: the Pittsburgh epidemiology of diabetes complications study cohort Diabetes 2012 61 2987 2992 10.2337/db11-1625 22851572 PMC3478551

[B5] Laing SP Swerdlow AJ Slater SD Burden AC Morris A Waugh NR Gatling W Bingley PJ Patterson CC Mortality from heart disease in a cohort of 23,000 patients with insulin-treated diabetes Diabetologia 2003 46 760 765 10.1007/s00125-003-1116-6 12774166

[B6] Skrivarhaug T Bangstad HJ Stene LC Sandvik L Hanssen KF Joner G Long-term mortality in a nationwide cohort of childhood-onset type 1 diabetic patients in Norway Diabetologia 2006 49 298 305 10.1007/s00125-005-0082-6 16365724

[B7] Orchard TJ Costacou T Kretowski A Nesto RW Type 1 diabetes and coronary artery disease Diabetes Care 2006 29 2528 2538 10.2337/dc06-1161 17065698

[B8] DCCT The diabetes control and complications trial N Engl J Med 1993 329 977 986 8366905 10.1056/NEJM199309303291410

[B9] Diabetes Control and Complications Trial/Epidemiology of Diabetes Interventions and Complications (DCCT/EDIC) Study Research Group Intensive diabetes treatment and cardiovascular disease in patients with diabetes type 1 N Engl J Med 2005 353 2643 2653 16371630 10.1056/NEJMoa052187 PMC2637991

[B10] Purnell JQ Hakanson JE Marcovina SM Steffes MW Cleary PA Brunzell JD Effect of excessive weight gain with intensive therapy of type 1 diabetes on lipid levels and blood pressure JAMA 1998 280 140 146 10.1001/jama.280.2.140 9669786 PMC2622729

[B11] Ceriello A Sudhesh K Piconi L Esposito K Giugliano D Simultaneous control of hyperglycemia and oxidative stress normalizes endothelial function in type 1 diabetes Diabetes Care 2007 30 649 654 10.2337/dc06-2048 17327335

[B12] Orchard TJ Olson JC Erbey JR Williams K Forrest KY Smithline Kinder L Ellis D Becker DJ Insulin resistance-related factors, but not glycemia, predict coronary artery disease in type 1 diabetes Diabetes Care 2003 26 1374 1379 10.2337/diacare.26.5.1374 12716791

[B13] Gomes MB Cobas RA Nunes E Castro-Faria-Neto HC da Matta MF Neves R Plasma PAF–acetylhydrolase activity, inflammatory markers and susceptibility of LDL to in vitro oxidation in patients with type 1 diabetes mellitus Diabetes Res Clin Pract 2009 85 61 68 10.1016/j.diabres.2009.04.016 19464746

[B14] Grundy SM Benjamin IJ Burke GL Chait A Eckel RH Howard BV Mitch W Smith SC Jr Sowers JR Diabetes and cardiovascular disease: a statement for healthcare professionals from the American Heart Association Circulation 1999 100 1134 1146 10.1161/01.CIR.100.10.1134 10477542

[B15] van Vliet M Van der Heyden JC Diamant M Von Rosenstiel IA Schindhelm RK Aanstoot HJ Veeze HJ Overweight is highly prevalent in children with type 1 diabetes and associates with cardiometabolic risk J Pediatr 2010 156 923 929 10.1016/j.jpeds.2009.12.017 20223481

[B16] Schwab KO Doerfer J Marg W Schober E Holl RW On behalf of the DPV Science Initiative and the Competence Network Diabetes Mellitus Characterization of 33488 children and adolescents with type 1 diabetes based on the gender-specific increase of cardiovascular risk factors Pediatr Diabetes 2010 11 357 363 10.1111/j.1399-5448.2010.00665.x 20624248

[B17] Petitti DB Klingensmith GJ Bell RA Andrews JS Dabelea D Imperatore G Marcovina S Pihoker C Standiford D Waitzfelder B Mayer-Davis E SEARCH for Diabetes in Youth Study Group The SEARCH for diabetes in youth study J Pediatr 2009 155 668 672 10.1016/j.jpeds.2009.05.025 19643434 PMC4689142

[B18] Rodriguez BL Dabelea D Liese AD Fujimoto W Waitzfelder B Liu L Bell R Talton J Snively BM Kershnar A Urbina E Daniels S Imperatore G SEARCH Study Group Prevalence and correlates of elevated blood pressure in youth with diabetes mellitus: the SEARCH for diabetes in youth study J Pediatr 2010 157 245 251 10.1016/j.jpeds.2010.02.021 20394942

[B19] Gomes MB de Mattos Matheus AS Calliari LE Luescher JL Manna TD Savoldelli RD Cobas RA Coelho WS Tschiedel B Ramos AJ Fonseca RM Araujo NB Almeida HG Melo NH Jezini DL Negrato CA Economic status and clinical care in young type 1 diabetes patients: a nationwide multicenter study in Brazil Acta Diabetol 2012 doi:http://dx.doi.org 10.1007/s00592-012-0404-3 10.1007/s00592-012-0404-3 22688518

[B20] Gomes MB Coral M Cobas RA Dib SA Canani LH Nery M de Freitas MC Faria M Felício JS da Silva SC Pedrosa H Costa e Forti A Rea RR Pires AC Montenegro Junior R Oliveira JE Rassi N Negrato CA Prevalence of adults with type 1 diabetes who meet the goals of care in daily clinical practice: a nationwide multicenter study in Brazil Diabetes Res Clin Pract 2012 97 63 70 10.1016/j.diabres.2012.02.008 22397904

[B21] Gomes MB Tannus LR Cobas RA Matheus AS Dualib P Zucatti AT Cani C Guedes AD Santos FM Sepulveda J Tolentino M Façanha MC Faria AC Lavigne S Montenegro AP Rodacki M de Fatima Guedes M Szundy R Cordeiro MM Santos PT Negrato CA Brazilian Type 1 Diabetes Study Group (BrazDiab1SG) Determinants of self-monitoring of blood glucose in patients with type 1 diabetes: a multicenter study in Brazil Diabetic Med 2013 doi:10.1111/dme.12236 10.1111/dme.12236 23721292

[B22] American Diabetes Association Clinical practice recommendations Diabetes Care 2013 34 S11 S66

[B23] Kuczmarski RJ Ogden CL Grummer-Strawn LM Flegal KM Guo SS Wei R Mei Z Curtin LR Roche AF Johnson CL CDC growth charts: United States Adv Data 2000 314 1 27 11183293

[B24] Friedwald WT Levy R Fredrickson DS Estimations of serum low density lipoprotein cholesterol without use of preparative ultracentrifuge Clin Chem 1972 18 499 502 4337382

[B25] Brazilian Diabetes Society Tratamento de crianças e adolescentes com diabetes mellitus tipo 1. **Brazilian Diabetes Society Guidelines 2013-2014** 78 99

[B26] Instituto Brasileiro de Geografia e Estatística (IBGE) Censo 2000 [article online] Available from http://www.ibge.gov.br/censo. Accessed August ,29th, 2008

[B27] Malerbi DA Franco LJ The Brazilian Cooperative Group on the Study of Diabetes Prevalence Multicenter study of the prevalence of diabetes mellitus and impaired glucose tolerance in the urban Brazilian population aged 30-69 yr Diabetes Care 1992 15 1509 1516 10.2337/diacare.15.11.1509 1468278

[B28] ABEP Brazilian economic classification criteria, 2010 [article online], 2010 Available from http://www.abep.org/novo/Content.aspx?SectionID = 84. Accessed August 2008

[B29] Ziegler R Heidtmann B Hilgard D Hofer S Rosenbauer J Holl R for the DBV-Wiss-Initiative Frequency of SBGM correlates with HbA1c and acute complications in children and adolescents with type 1 diabetes Pediatr Diabetes 2010 12 11 17 20337978 10.1111/j.1399-5448.2010.00650.x

[B30] Hood KK Peterson CM Rohan JM Drotar D Association between adherence and glycemic control in pediatric type 1 diabetes: a meta-analysis Pediatrics 2011 124 1171 1179 10.1542/peds.2009-0207 19884476

[B31] Pihoker C Badaru A Anderson A Morgan T Dolan L Dabelea D Imperatore G Linder B Marcovina S Mayer-Davis E Reynolds K Klingensmith GJ SEARCH for Diabetes in Youth Study Group Insulin regimens and clinical outcomes in a type 1 diabetes cohort: the SEARCH for diabetes in youth study Diabetes Care 2013 36 27 33 10.2337/dc12-0720 22961571 PMC3526205

[B32] Paris CA Imperatore G Klingensmith G Petitti D Rodriguez B Anderson AM Schwartz ID Standiford DA Pihoker C Predictors of insulin regimen and impact outcomes in youth with type 1 diabetes: the SEARCH for diabetes in youth study J Pediatr 2009 155 183 189 10.1016/j.jpeds.2009.01.063 19394043

[B33] Urbach SL LaFranchi S Lambert L Lapidus JA Daneman D Becker TM Predictors of glucose control in children and adolescents with type 1 diabetes mellitus Pediatr Diabetes 2005 6 69 74 10.1111/j.1399-543X.2005.00104.x 15963032

